# Experimental mechanical strain measurement of tissues

**DOI:** 10.7717/peerj.6545

**Published:** 2019-03-07

**Authors:** Lingwei Huang, Rami K. Korhonen, Mikael J. Turunen, Mikko A.J. Finnilä

**Affiliations:** 1 Department of Applied Physics, University of Eastern Finland, Kuopio, Finland; 2 Research Unit of Medical Imaging, Physics and Technology, University of Oulu, Oulu, Finland; 3 Medical Research Center Oulu, Oulu University Hospital, Oulu, Finland

**Keywords:** Mechanical strain, Tissue, Mechanical loading, Deformation, Biomechanics

## Abstract

Strain, an important biomechanical factor, occurs at different scales from molecules and cells to tissues and organs in physiological conditions. Under mechanical strain, the strength of tissues and their micro- and nanocomponents, the structure, proliferation, differentiation and apoptosis of cells and even the cytokines expressed by cells probably shift. Thus, the measurement of mechanical strain (i.e., relative displacement or deformation) is critical to understand functional changes in tissues, and to elucidate basic relationships between mechanical loading and tissue response. In the last decades, a great number of methods have been developed and applied to measure the deformations and mechanical strains in tissues comprising bone, tendon, ligament, muscle and brain as well as blood vessels. In this article, we have reviewed the mechanical strain measurement from six aspects: electro-based, light-based, ultrasound-based, magnetic resonance-based and computed tomography-based techniques, and the texture correlation-based image processing method. The review may help solving the problems of experimental and mechanical strain measurement of tissues under different measurement environments.

## Introduction

Strain is an important mechanical factor that affects strain-associated biological function. Loading of an organ is transferred progressively to smaller scales in a multiscale manner, first to tissues and then to pericellular and cellular levels. At the tissue level, the strains are distributed to the micro- and nanostructure. The structures are optimized to withstand the strains and loads experienced by the tissues. Collagen, a major protein in tissues, contributes to the mechanical properties including ductility under tension but also compressive behavior in many tissues, while mineral crystals provide the rigidity in mineralized tissues, e.g., bone. At the cellular level, mechanical strain can lead to biochemical signal transduction modulating tissue metabolism, and finally influence the function of the organism (Jacobs, Temiyasathit & Castillo, 2010). Mechanical strain can then modulate activity and gene expression in cells leading to altered structure and function of tissues.

Mechanical loading exists almost everywhere in cells, tissues, and their matrix components under physiological conditions, which deform under these conditions. Mechanostat theory, a tissue-level negative feedback system, has two mechanical strain thresholds which determine tissue strength by switching on and off the biological mechanisms (Frost, 2004). The strain in tissues induced by mechanical loading can produce fluid flow which influences cells inside the tissues, just as we reviewed before about osteoblasts (Huang et al., 2015). Therefore, the judgment of mechanical deformation (i.e., displacement or mechanical strain) is critical to understand functional changes in cells and biological tissues under physiological loading, and to elucidate basic relationships between mechanical strain and the tissue conditions.

At present, texture analysis methods have allowed strain measurement from various imaging methods including optical imaging, magnetic resonance imaging (MRI), X-ray microscopy, and ultrasound (US) imaging, and they are attractive and alternative for traditionally used strain gauge techniques. Recently, reviews on strain characterization include for example *in vivo* strain measurement of bone (Al Nazer et al., 2012; Yang, Bruggemann & Rittweger, 2011) and ligaments (Fleming & Beynnon, 2004) in humans and in-plane strain measurement with digital image correlation (DIC), current methods on strain characterization on bone (Grassi & Isaksson, 2015), and the feasibility of non-Doppler US methods with speckle tracking for fetal myocardial strain evaluation (Germanakis & Gardiner, 2012). However, these reviews are limited on strain evaluation of one or a few techniques or tissues. Though they can help improving our understanding of the application of some strain assessment techniques in certain tissues, they do not provide a complete review of the state-of-art strain characterization techniques in tissues in general. To provide a relatively comprehensive understanding of various tissue strain measurements and provide a convenient way in choosing a suitable experimental methodology, we reviewed the mechanical strain measurement techniques from six aspects: electro-based, light-based, ultrasound-based (US-based), magnetic resonance-based (MR-based) and computed tomography-based (CT-based) techniques, and the texture correlation-based (TC-based) image processing method. The review may help interpreting and debugging problems and challenges of mechanical strain estimation in biological tissues during experiments and clinical applications. Furthermore, this review might aid in choosing adequate mechanical strain estimation tools for a study based on the listed pros and cons.

## Survey Methodology

To search the available reports on the experimental and mechanical strain measurement of biological tissues, a standardized search strategy was conducted to survey studies indexed in PubMed and Web of Science databases. The search terms were selected as “strain”, “deformation”, and “tissue”, together with “measurement”, “evaluation”, and “quantification”. We strictly searched for publications focusing on the experimental and mechanical strain measurement of tissues that represent the state-of-art methodology and that are continuously used in current research. Research published before November 2018 were collected based on our criteria. After excluding the strain measurement of artificial materials and the strain measurement in cell, molecule or atom level, and removing the old fashioned or modeling of strain, 143 articles were selected in our manuscript, among which, there were 10 physiological mechanical strain related introduction articles, 37 electro-based mechanical strain measurement articles, 12 light-based mechanical strain measurement articles, 21 US-based mechanical strain measurement articles, 16 MR-based mechanical strain measurement articles, 12 CT-based mechanical strain measurement articles, 42 TC-based image processing articles. In seven articles, different strain measurement techniques were compared or combined.

## Summary of the Mechanical Strain Measurement

Six types of tissue strain assessment approaches including electro-based, light-based, US-based, MR-based and CT-based techniques, together with the TC-based image processing method were analyzed. The mechanisms and possibilities of different techniques for the strain measurement in various tissues have been summarized in Table 1 and further explained in the following chapters.

**Table 1:** Mechanisms of strain measurement for different methods and their possible applications

Type	Approach	Mechanism of mechanical strain evaluation	Strain range	Test	Target tissue	Reference examples
Electro-based	Strain gauge	The deformation of tissues induces the electrical signal changes, which can be converted into strain values of the tissues.	10∼10^7^µε	Discrete *In vivo* *Ex vivo*	Bone Cartilage	Takano et al. (1999)Szivek, Anderson & DeYoung (1997)Pintar et al. (1995) Rolf et al. (1997) Markolf et al. (1998)
	Strain transducers				Ligament Tendon Muscle	
Light-based	Microscopy camera	The relative strain is assessed by comparing the images before and after the tissue deformation.	10^2^∼10^6^µε	Serial *Ex vivo*	Cartilage Ligament Tendon Nerve Blood vessel	Moo et al. (2018) Wright et al. (1996)Bartell et al. (2015) Teo, Dutton & Han (2010) Butler et al. (1990) Squire, Rogers & Edelman (1999)
US-based	Tissue Doppler imaging	The strain is calculated from US images of the tissues, according to the Doppler effect (frequency shift) of the reflected US incited by the deformation of tissues.	10^3^∼10^6^µε	Serial *Ex vivo In vivo*	Myocardial wall Gastric wall Vascular wall	Perk, Tunick & Kronzon (2007) O’Neill et al. (2007) Ling, Zheng & Patil (2007) Amundsen et al. (2006) Gilja et al. (2002) Liang, Zhu & Friedman (2008) Wilson, Press & Zhang (2009b) Bihari et al. (2013)
	US elastography	The strain of tissues is assessed by the correlation of the pulsed US echo signals in windows before and after tissue deformation.		Serial *In vivo* *Ex vivo*	Myocardial wall Gastric wall Vascular wall Cartilage Tendon Ligament	
	Speckle tracking echocardiography	Strain is quantified from changed reflection US interference patterns in the US images during the deformation of the tissues.		Serial *In vivo* *Ex vivo*		
Magnet-based	Tag tracking MRI	The applied magnetization tags in the tissues change with the deformation of tissues and strain messages can be extracted from the changed images of tags.	10^2^∼10^6^µε	Serial *In vivo* *Ex vivo*	Myocardium Bone Cartilage Tendon Ligament Liver Brain	Axel (1997) Al Nazer et al. (2008) Sutter et al. (2015) Sheehan & Drace (2000) Mannelli et al. (2012) Hirsch et al. (2013)
	Elastography MRI	Strain is assessed from changed signal patterns in MR images obtained from the tissues before and after their deformation.		Serial *In vivo* *Ex vivo*		
CT-based	CT	Strain values are acquired from the changes of reconstructed 3D structure of tissues before and after deformation.	10∼10^4^µε	Serial *In vivo* *Ex vivo*	Bone Cartilage Heart Calcified cartilage Blood vessel	Novitskaya et al. (2014) Halonen et al. (2014) Pierce et al. (2016) Boekhoven et al. (2014) Gustafsson et al. (2018)
TC for image processing	DIC	Strain is evaluated by tracking the subsets including markers or speckles on the surface of tissues.	10^2^∼10^4^µε	Serial *Ex vivo*	Bone Blood vessels Other tissues with marked surface	Hussein, Barbone & Morgan (2012) Sheehan & Drace (2000) Bey et al. (2002a) Cyganik et al. (2014)
	DVC	Strain is evaluated by tracking image subsets by tracking the natural pattern in the tissues.		Serial *In vivo* *Ex vivo*	All tissues with specific structure features	Bay (1995) McKinley, English & Bay (2003) Bay et al. (1999) Toh et al. (2006)

## Electro-based Mechanical Strain Measurement

Electro-based mechanical strain measurement techniques are widely used for the evaluation of tissue deformations. Especially, the strain gauge technique is commonly applied as a golden standard in the mechanical strain measurement of tissues. In the following parts, all the electro-based mechanical strain measurement techniques are treated as “strain sensors”.

### The principle of electro-based mechanical strain measurement

**Figure 1:**
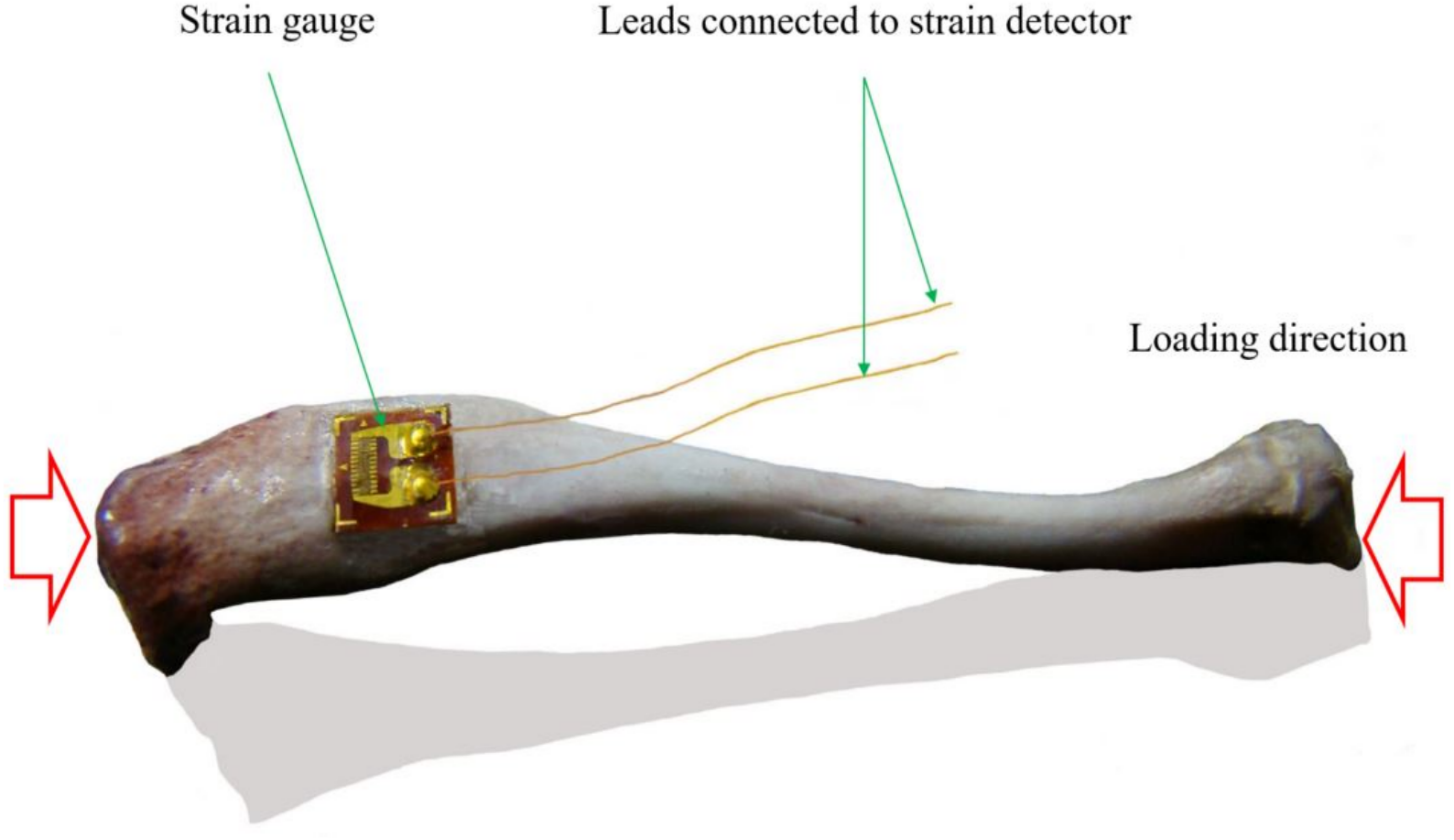
Schematic view of the axial strain measurement of tibia with single strain gauge. When bone deforms, the attached strain gauge will deform, and the embedded metal strain resistance wire will deform too, resulting in the resistance change of the metal strain resistance wire and finally resulting in changed output signals, and the changed signals can be transferred into strain using a strain detector (this figure was generated from a rat tibia by a Lingwei Huang).

This technique is built on the electrical resistance change of the strain sensors. When a tissue deforms, the electrical resistance of strain sensors inserted or attached to the tissue will change, which results in changed output electrical signal. The electrical signal, which is proportional to the tissue deformation, is amplified and detected with a signal acquisition device (e.g., MP160WSW, BIOPAC, USA) and then the signal is collected with a related software in a computer as digital data. Finally, the collected data is converted into mechanical strain value. There are various types of strain gauges including bare strain gauges (without coating layers), coated strain gauges, strain transducers, instrumented strain gauges, and differential reluctance transducers. Bare strain gauges are extensively applied for *ex vivo* or temporarily *in vivo* mechanical strain measurement of hard tissues including bone (Fig. 1). Coated strain gauges consisting of hydroxyapatite-coated (HA-coated), calcium phosphate ceramic-coated (CPC-coated) and resin coated strain gauges, are verified and available for *in vivo* mechanical strain measurement of tissues, especially bone. Strain transducers are usually composed of polymers and are universally used for the mechanical strain measurement of soft tissues such as ligament, nerve and cartilage. Differential reluctance transducers are created on the varying magnetic flux with their deformation caused by tissues and the altered magnetic flux will be transformed into electrical signals for mechanical strain assessment of surrounding tissues. Instrumented screws can be used to record mechanical strain and they are widely applied for the mechanical strain evaluation of hard tissues, especially those with irregular shapes.

### The application of electro-based mechanical strain measurement

#### Strain gauge

*Ex vivo* mechanical strains around natural teeth with prostheses and at femoral necks and metatarsals in human cadavers were evaluated with strain gauges bonded on bone during mechanical loading (Cehreli et al., 2005; Enns-Bray et al., 2016; Fung, Loundagin & Edwards, 2017). In one *ex vivo* experiment, four triple strain gauge rosettes were used to measure the mechanical strain of metacarpus and distal row of horse carpal bones (Les et al., 1998). Rosette strain gauges were also used to study the mechanical strain shielding of plates in sheep tibias with rosette strain gauges fixed onto the anterior and posterior aspects of the tibias (Gautier, Perren & Cordey, 2000). In another study, three strain gauges were placed around the mid-shaft of the radius of foxhounds to find the relationship of peak functional and mechanical strains of bone (Takano et al., 1999). Because of electrical signal disturbance caused by intracorporal environment, common strain gauge applications without the protection of coat layers are limited. Dielectric material coated strain gauges and instrumented strain gauges are developed to reduce the influence of *in vivo* environment on the transduction of electrical signals in strain gauges and are widely utilized for *in vivo* mechanical strain measurement.

#### Coated strain gauges

The bonding of HA-coated strain gauges and bone was analyzed with cantilever bending test by comparing the strain change after 6–7 weeks’ adhesion, and 70–100% bonding was found between HA-coated strain gauges and rat femur (Wilson et al., 2009a). Another study in greyhound femur showed well-bonded and sensing accuracy after 4-month implant of the coated strain gauges (Szivek et al., 1990). CPC-coated strain gauges were also exploited to accurately measure bone mechanical strain *in vivo* during exercises (Rabkin et al., 2001). *In vivo* and *ex vivo* mechanical strain evaluation of the proximal femora of dogs at a series of gait speeds using CPC-coated strain gauges showed that mechanical strain patterns were similar and peak mechanical strains were the same in the following 2 weeks (Szivek, Anderson & DeYoung, 1997). Resin coated rosette strain gauges could also be exploited to measure surface mechanical strain on the dorsomedial cortex of the third metacarpal bones in six adult horses during treadmill exercise (Davies, Mccarthy & Jeffcott, 1993).

#### Strain transducers

In one study, the strain transducers were implanted into medial and lateral ligaments of the ankles harvested from human cadavers to measure mechanical strain changes of the ankle ligaments (Ozeki et al., 2002). In another study, the mechanical strain in the anteromedial band of the anterior cruciate ligament of thirteen fresh frozen cadaveric knee specimens was also measured using transducers with barbed prongs inserted into the anteromedial band to record local elongation of the instrumented fibers (Markolf et al., 1998). Further, Pozzi *et al.* inserted subminiature differential variable reluctance transducers into the periphery of the caudal pole of the menisci of adult dogs to measure the mechanical strain of the caudal region before and after loading under meniscectomies (Pozzi, Tonks & Ling, 2010). Also in one human cadaver study, differential reluctance transducers have been placed in the superficial peroneal nerve in sixteen lower-extremity specimens to measure mechanical strain *in situ* to find the strain variation in the intact and the sectioned anterior talofibular ligaments during a simulated inversion sprain (O’Neill et al., 2007). In another human study, the mechanical strains of the anterior cruciate ligaments were measured using a differential variable reluctance transducer attached, during closed kinetic chain exercises (Heijne et al., 2004) and simulated movement (Hacker et al., 2018).

#### Instrumented strain gauges

In human studies, surgical staples with two strain gauges were inserted through skin incision into the cortical bone of tibia to measure local bone deformation *in vivo* under static conditions (Rolf et al., 1997), and during exercises (Milgrom et al., 2000a; Milgrom et al., 2000b). In clinical practice, strain gauges were attached to the fixation rod of 500 bone fracture patients with external bone fixator to measure the rod deformation when raising the limb to a given angle in the healing process (Burny et al., 1984), so that the bone deformation beneath the fixator could be evaluated. Linear relationship was found between the calculated mechanical strain at the bone surface and the strain measured by the instrumented bone staples, suggesting instrumented staples’ effectiveness for local bone deformation measurement (Ekenman et al., 1998). Even so, higher mechanical strain was observed at the bone-screw interface in the end regions of an anterior solid rod construct during lateral bending (Spiegel et al., 2000).

Electro-based techniques are widely used as principle methods for mechanical strain measurement. Nevertheless, the techniques are limited to superficial or simple mechanical strain measurement of tissues. Also, the precision of the measurement is largely dependent on the quality of the strain sensors themselves and their attachment, and the attached strain sensors can also affect the deformation of a sample to some degree.

## Light-based Mechanical Strain Measurement

Light-based mechanical strain measurement is a non-contacting strain evaluation method.

### The principle of light-based mechanical strain measurement

Light-based mechanical strain measurement is established on extracting tissue strains from deformation induced change of optical information (e.g., image datasets). During the deformation, the markers and special features in tissues are tracked and recorded with laser beam and image recorders respectively. Then, the markers’ movement or the features’ change is evaluated between those before and after deformation. Finally, mechanical strains are extracted from the changed markers’ positions or features’ characteristics according to image processing algorithms.

### The application of light-based mechanical strain measurement

High speed camera and confocal laser scanning microscopy can be used to measure localized deformation of cartilage or other tissues via comparing the alterations of gathered pictures. Bulk mechanical strain was recently calculated using the image taken at peak indentation and the image before deformation (Bartell et al., 2015). Confocal laser scanning microscopy combined with image analysis could also measure global, local axial and transverse mechanical strains of cartilages *in situ* (Fick et al., 2016; Fick et al., 2015; Turunen et al., 2013), by tracking markers in the tissues, i.e., cells (Mansfield, Bell & Winlove, 2015). Analogously, the surface deformation of human ulnar collateral ligament was studied by tracking the artificial markers fixed on the tissue surface during mechanical testing (Smith et al., 2019). Recently, a novel method was used to quantify three dimensional (3D) tissue strain of intact cartilage in *ex vivo* pig knees at sub-micrometer resolution (Moo et al., 2018)*.* In this microscopic technique, a 3D grid is imprinted into fibrous tissue and strain can be quantified by imaging the grid deformation at various strain/stress levels.

## US-based Mechanical Strain Measurement

Ultrasound is popularly adopted for non-destructive mechanical strain estimations of tissues both *in vivo* and *ex vivo*. US-based mechanical strain measurement can be mainly divided into tissue Doppler imaging (tissue Doppler echocardiography), US elastography and speckle tracking echocardiography. US imaging or sonography is often exploited in the mechanical strain assessment of tissues.

### The principle of US-based mechanical strain measurement

In ultrasonic imaging, US is emitted into tissues and the echoed US is recorded and displayed as images. By comparing the recorded images before and after the deformation of the tissues, strain information can be extracted. Familiarly, pulsed US is employed in US imaging due to its high signal-to-noise ratio. There are various US elastography techniques that also allow strain quantification. One benefit of US is that besides imaging deformation caused by external loading, also deformation caused by physiological processes or US radiation can be utilized for elastography. The mechanical strain of tissues is assessed by the correlation of the pulsed US echo signals in windows before and after tissue deformation. For speckle tracking echocardiography, the reflected US produces constructive and destructive interferences, and the interferences (i.e., speckles) will redistribute during the deformation of the tissues, which leads to relative displacements of the interference patterns in the US images, and the displacements are tracked for the assessment of tissue deformations. For tissue Doppler imaging, US with a high frequency creates an image of the tissues, according to the Doppler effect (frequency shift) of the reflected US incited by the deformation of tissues.

### The application of US-based mechanical strain measurement

US can be used in mechanical strain measurement by Doppler-based or Non-Doppler-based techniques. The Doppler-based technique is angle dependent, whose measurement can be only done along the orientation of ultrasonic beam. While Non-Doppler strain imaging is angle independent and can be used for clinical mechanical strain measurement of myocardial wall (Perk, Tunick & Kronzon, 2007).

*In vitro* research on bovine articular cartilage showed that US speed in the sample was highly correlated to the applied mechanical strain in a quadratic relation and ultrasound speed changed by 7.8% when the applied compression strain reached 20% (Ling, Zheng & Patil, 2007), which showed close relationship between strain value and the change in US speed. Speckle tracking echocardiography was validated as a method for *in vivo* angle-independent measurement of regional myocardial strain of humans (Amundsen et al., 2006). Strain rate imaging, a method in echocardiography, was employed for radial deformation measurement of human left ventricle (Herbots et al., 2004). Similarly, two dimensional (2D) myocardial deformation of human left ventricles was estimated using the angle-independent myocardial elastography (a radio-frequency based speckle tracking technique) and the tagged MRI, and the deformation estimations by the two methods were in good agreement with each other (Lee et al., 2008). Multidimensional radio-frequency echo phase matching method was also applied to measure deformations in the lateral and axial directions (Sumi, 2007). In addition, transabdominal strain rate imaging, a Doppler US method was utilized to explore the mechanical strain of the muscle layers within the gastric wall during gastric contractions in humans (Gilja et al., 2002).

Additionally, radiofrequency US-based imaging techniques were devoted for radial and longitudinal mechanical strain measurement of the sub-endocardial, mid-wall and sub-epicardial layers of tissues from healthy and infarcted regions in five pigs (Van Slochteren et al., 2014). Intravascular US elastography was enhanced by developing a 2D mechanical strain estimation method to acquire the strain tensor that reflects mechanical strains in any direction in the cross-section of artery wall (Liang, Zhu & Friedman, 2008). Ultrasonography imaging in combination with computational methods was applied for the mechanical strain measurement of human Achilles tendons (Peltonen et al., 2013; Stokes et al., 2010) and quadriceps tendons (Wilson, Press & Zhang, 2009b) *in vivo*, and rabbit Achilles tendons *ex vivo* (Kuo et al., 1999). The procedure varies among different US systems for mechanical strain measurement. Investigation of two echocardiograms of patients from two different commercial US systems showed that post-processing is the most important determinant in inter-vendor variation (Negishi et al., 2013).

Universally, US-based mechanical strain measurement is a promising method for simple strain assessment of tissues, especially clinically. 3D strain inside the tissues, especially for heterogeneous or irregular tissues, cannot be evaluated, though 3D echocardiography has been devoted for 3D cardiac deformation estimation, utilizing an algorithm concerning a transversely isotropic linear elastic model (Papademetris et al., 2001). A real-time 3D speckle tracking US was established to explore the local wall strain of the whole abdominal aortic aneurysm in patients *in vivo* (Bihari et al., 2013). US tomography approach (echo-computed tomography) could be also applied to estimate local deformations of human carotid using a 2D mechanical strain algorithm, and 3D radial mechanical strain data was reconstructed (Boekhoven et al., 2014).

## MR-based Mechanical Strain Measurement

Magnetic resonance based mechanical strain measurement techniques mostly focus on the mechanical strain measurement with MRI, though another system presented its capability in monitoring the real-time deformation of intracranial brain during impact-induced brain injury of rats through detecting the changing magnetic field produced by the movement of implantable soft magnet (Song et al., 2015). MRI can be divided into MR elastography and MR tag tracking in accordance with the integrated techniques. Because of no negative effects reported, MRI is looked as a reliable and secure way for the mechanical strain evaluation of brain, heart, and other important tissues where the tissue strains are difficult to be investigated with other mechanical strain measurement techniques without side effect on the organisms *in vivo*.

### The principle of MR-based mechanical strain measurement

There are specific sequences (e.g., DENSE) for mechanical strain imaging. Alternatively, deformation of tissues can be assessed by following the magnetization tags in the tissues or evaluating the changes of signal patterns with texture correlation (TC) image processing methods.

### The application of MR-based mechanical strain measurement

MRI is based on magnetization tags (temporary features in tissues produced by a special pulse sequence and moving with the tissues) tracing and mechanical strain-induced phase shifts in tissues (Axel, 1997) which can be translated into the deformation of the tissues. MRI with tagging is generally used for noninvasive myocardial strain assessment with acceptable accuracy (Lima et al., 1993). The assessment of cardiac deformation by cardiovascular MRI combined with feature tracking has been validated feasible in children (Andre et al., 2016). Cardiac MRI was developed for measuring peak systolic circumferential strain (Simonetti & Raman, 2010) and intramyocardial strain (Axel, 1997). In addition, a transmural gradient in the mechanical strain of a normal dog heart was detected with MRI together with multispectral radio-frequency pulses produced tagging grids for high-resolution mechanical strain estimation (McVeigh & Zerhouni, 1991). With magnetization tags, MRI was also used to quantify the cardiac induced liver strain and the head’s angular acceleration produced brain strain in humans (Chan et al., 2018; Mannelli et al., 2012). Motion-sensitive phase contrast MRI was applied for the measurement of volumetric strain of brain (Hirsch et al., 2013) and patellar tendons (Sheehan & Drace, 2000) in humans as well. Using MRI and image registration techniques, *in vivo* 3D deformation of cervical spinal cord in rat was quantified (Bhatnagar et al., 2016). MRI could also be used to measure local tibiofemoral cartilage strains in response to a dynamic hopping activity (Sutter et al., 2015) and the dynamic mechanical strains in tibia during human locomotion (Al Nazer et al., 2008), combined with an iterative closest point technique (i.e., MR tag tracking) and a flexible multibody approach (i.e., MR elastography) respectively. Besides, MR-based technique was utilized to quantify intratendinous mechanical strains in cadaveric shoulder specimens at superior, middle, and inferior locations across the regions where most rotator cuff tears occur clinically (Bey et al., 2002a).

## CT-based Mechanical Strain Measurement

Computed tomography (CT) is a very common technique in 3D structure characterization of tissues. The main parts of the CT are X-ray sources, detectors and associated software for reconstruction and visualization, and possibly for structural and mechanical strain analysis.

### The principle of CT in mechanical strain measurement

First, projection images of the tissues before and after deformation are acquired. This can be a time consuming process especially with a high-resolution experimental system. Then, the original images are reconstructed and modeled into 3D structures of the tissues (one of the tissues before deformation and one of the tissues after deformation). Finally, the strain information of the tissues can be evaluated through comparing the tissues’ structure before and after deformation.

### The application of CT in mechanical strain measurement

With contrast media, a novel cone beam CT-scanners combined with an analyzing software was applied to study the mechanical strain of articular cartilage in human knees during static loading (Halonen et al., 2014). Similarly, micro-computed tomography (micro-CT) scanner could be applied to evaluate mechanical strain in human cadaveric meniscal tissues by tracking small Teflon markers implanted (Kolaczek et al., 2016) and assess large deformations of ovine hearts by tracking fiducial markers applied (Pierce et al., 2016). The creep deformation of human trabecular bone samples from proximal tibia was assessed as well by analyzing the tissue architecture both before and after creep using micro-CT imaging (Novitskaya et al., 2014).

## TC-based Image Analysis of Mechanical Strain

Texture correlation is a widely utilized image processing method for the characterization of changes in structure, i.e., tissue deformation. This strain evaluation method in tissues depends on the comparison of images from the tissues before and after deformation.

### DIC

DIC is a TC-based method for non-contact, superficial deformation measurement (Kahnjetter & Chu, 1990) and can be used basically with any imaging modality. This method is increasingly used for *in vitro* set-ups (Shelton & Katz, 1991), and is particularly suitable for biological applications in view of its accurate mechanical strain measurement in inhomogeneous, anisotropic, non-linear materials, such as mandible (Tanasic et al., 2012).

#### The principle of DIC

**Figure 2:**
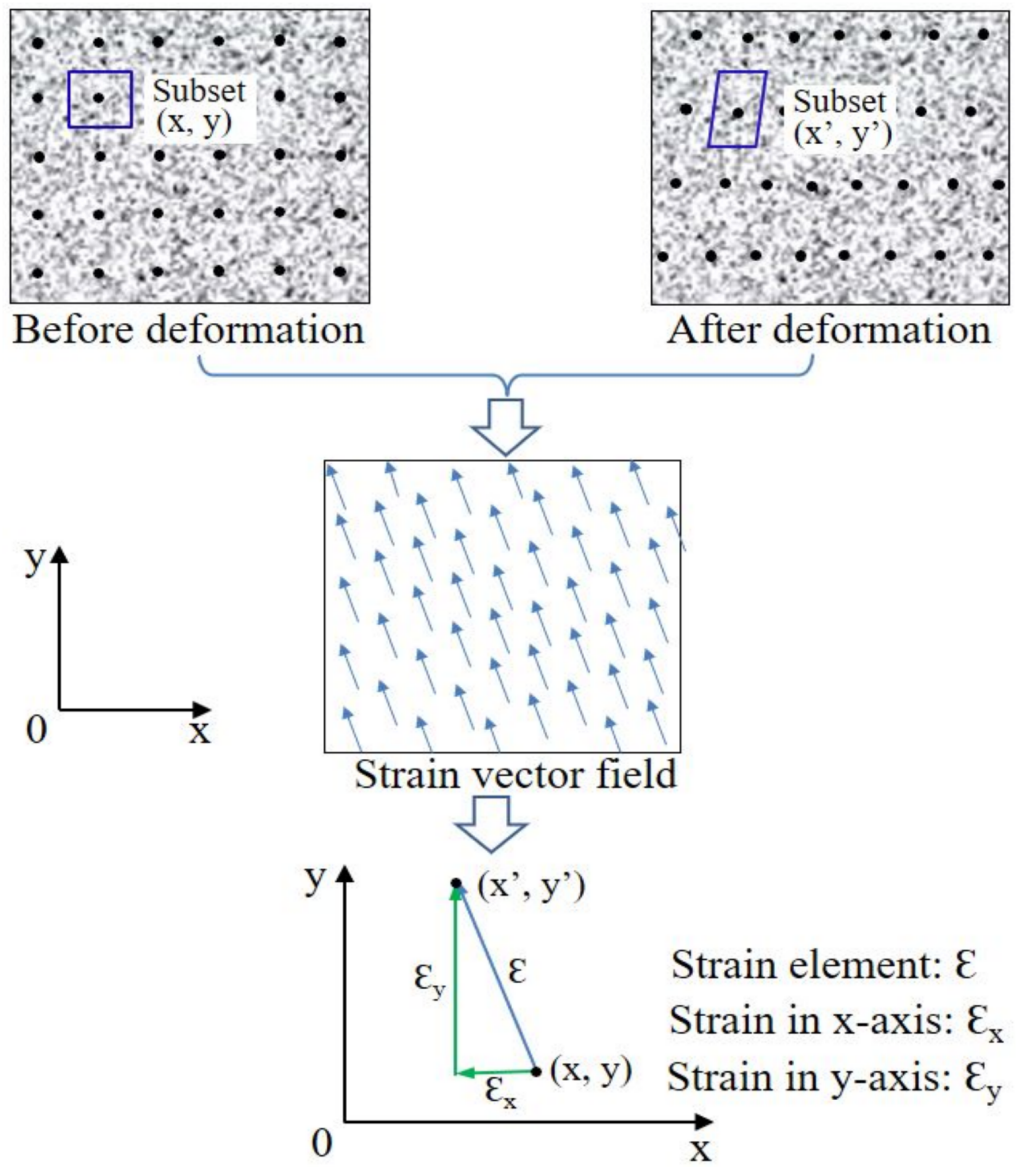
Principle of 2D strain measurement of region of interest in samples using DIC. Comparing the target and reference regions (consisting of subsets with speckles (black points in sample images) inside) of the sample, the varied characteristic features can be acquired and then converted into strain. Adapted from Dai et al. (2015).

DIC is an appropriate image processing method for assessing mechanical strain distribution throughout the structures with complex geometries (Rodriguez et al., 2004). The basic principle of DIC is to co-register the same physical points between the two images recorded before and after deformation (Fig. 2). A square reference subset centered at the interrogated point in the reference image is chosen and used to track its corresponding location motion. The displacement field can be determined by the calculations with a derivative algorithm. To evaluate the similarity degree between the reference and target subsets, a certain correlation criterion should be defined in advance (Dai et al., 2015). In general, zero-mean cross correlation criterion (Pan et al., 2009) is used. In some other similar methods, high contrast markers are sprayed onto the surface of the sample and observed with cameras during loading. The entire field of view is divided into a number of unique correlation areas, or ‘facets’, which typically contain a square subset of pixels. The characteristic features of the speckle pattern in the facets will be tracked during loading and the modified features provide a progressive measurement of deformation (Tanasic et al., 2012).

#### The application of DIC in mechanical strain measurement

2D image processing DIC combined with a high-quality imaging device is widely used for in-plane mechanical strain measurement as an effective and irreplaceable method (Pan et al., 2009). Combined with DIC, an experimental microextensometry approach has been developed to analyze the displacement and mechanical strain fields on the surface of the mature bovine cortical bone (Hoc et al., 2006). Besides, a series of images from ring porcine aorta surface were analyzed to quantify the local surface strain of vascular tissues subjected to ramped uniaxial loading (Bey et al., 2002a). Similarly, the displacement and full-field mechanical strain in human femoral heads and mouse forearms have been evaluated *ex vivo* using DIC, combined with camera images before and after mechanical loading (Begonia et al., 2015; Cyganik et al., 2014). The mechanical strain evaluation method of DIC is confined to 2D and some speckles under tracing may be missing, introducing challenges of evaluating the strain of tissues with curved surface.

To overcome the limitations of plain mechanical strain measurement of DIC, practical and effective 3D image processing approach DIC or digital volume correlation (DVC) has been developed for the strain measurement of tissues with both planar and curved surface (Pan et al., 2009). The uncertainties of DVC have been demonstrated acceptable combined with images from X-ray micro-CT and optical scanning tomography (Germaneau, Doumalin & Dupré, 2008).

### DVC

DVC is an extension of DIC and its applications have been reviewed previously (Bay, 2008). DVC is an image processing method that quantifies mechanical strains throughout the interior of the tissues, rather than simply on the surface, contributing to 3D mechanical strain evaluations. This technique relies on tracking the movement of structural features of tissues with subsets of voxels, rather than with subsets of pixels.

#### The principle of DVC

Similar to DIC, in DVC, voxels of the natural texture in biological tissues are utilized for the evaluation of mechanical strain fields between two consecutive digital images. This technique was developed and validated with six samples of trabecular bone from a single human femoral head, in which the motion of subsets within images was tracked and the displacement was acquired by comparing images of the initial (i.e., reference) and current (i.e., deformed) configuration (Bay, 1995).

#### The application of DVC in mechanical strain measurement

Mechanical strains of trabecular bone in the proximal and distal tibia and central vertebrae of human cadavers under mechanical loading were studied with DVC of radiographs from the bone with unloaded and different loaded conditions (Bay et al., 1999; McKinley & Bay, 2001; Gillard et al., 2014). DVC, together with contact radiographs of the tissues, was applied for mechanical strain measurement of specimens from proximal medial tibia of human cadavers under loading, and sharp rises in trabecular bone strain were found under increased subchondral bone defects (Brown, McKinley & Bay, 2002), complete meniscectomy (McKinley, English & Bay, 2003), or simulated subchondral stiffening (McKinley & Bay, 2003). Similarly, local mechanical strain fields in the mid-diaphyseal cortical bone of canine femurs (Kim, Brunski & Nicolella, 2005) and local distribution of minimum principal strain and maximum shear strain of intact (Yerby et al., 1998) and pedicle screw implanted thoracic spines of human cadavers (Toh et al., 2006) under mechanical loading were investigated with machine vision photogrammetry and digitized contact radiographs respectively, with DVC technique.

Presently, many micro-CT combined DVC have been presented for the mechanical strain quantification of tissues. DVC has been verified for 3D measurement of the deformation in *ex vivo* porcine lamina cribrosa, retrolaminar neural tissue and vertebrae under different mechanical loading conditions using images acquired from micro-CT (Coudrillier et al., 2016; Danesi, Tozzi & Cristofolini, 2016; Feola et al., 2017). In addition, the deformation in the spine of human and rats under mechanical loading was measured using deformable image registration algorithm combined with micro-CT (Choudhari et al., 2016; Hussein, Barbone & Morgan, 2012).

## Discussions and Conclusions

At present, various techniques consisting of optical imaging, MRI, X-ray scanning imaging, US imaging, and strain gauge technique, together with the TC-based image processing method, are familiarly taken for mechanical strain measurement of tissues comprising bone, tendons, ligaments, muscles, brain tissues and blood vessels. The characters and some applications of these techniques have been evaluated in Table 2.

**Table 2:** Information of the main methods for the mechanical strain measurement of tissues.

Technique	Dimension	Advantage	Disadvantage	Operation time	Image analysis
Strain gauge	2D 3D	Cheap; Few offline work	Invasive; Low anti-interference	Real-time	N/A
Strain transducers					
Microscopy camera	2D 3D	Cheap; Easy operation	Transparent or translucent samples	Range from minutes to hours	Marker-tracking algorithm
Tissue Doppler imaging	1D 2D	Cheap; Easy clinical application	Simple structure	Minutes	Baseband speckle- tracking algorithm; Registration algorithm
US elastography	2D				
Speckle tracking echocardiography					
Tag tracking MRI	3D	Safe and no side effect	Expensive; Time-consuming	Range from minutes to hours	Registration algorithm
Elastography MRI					
CT	3D	Relatively fast imaging; Relatively low cost	High contrast tissues needed; X-ray radiation	Range from seconds to hours	Registration algorithm

**Notes.**

a Most of the image analysis methods are TC-based; please see example references from Table 1.

The strain gauge method, one of the electro-based mechanical strain measurement techniques, is usually considered as a gold standard in bone strain measurement. The size of a typical strain gauge is several millimeters, staying in macroscopic level. In some conditions, the tissue surface is not big enough for the attachment of strain gauge. Miniature strain gauges can reduce the attaching area needed and have been employed for the mechanical strain measurement of a human cadaver cervical vertebral (Pintar et al., 1995) or condylar neck of miniature pigs during normal or simulated function (Marks et al., 1997). In clinical studies, miniature three-element rosette strain gauges were mounted on the medial and lateral surfaces of human patella and significant mechanical strain redistribution was found after graft removal (Steen et al., 1999). When the thickness of the epoxy film of the strain sensors cannot be ignored, it may influence the validity of measurement results of the mechanical strain. The mechanical behavior of thin film was measured and was found that the influence of the thin film can be ignored (Wang et al., 2014).

To minimize the influence of strain sensors on tissues’ mechanical strain survey, film sensors may be another good choice, though most of these techniques are applied in material engineering. Just like pure transducers (Bravo, Tersalvi & Tosi, 1992), the deposition of the strain gauge directly on the mechanical support by using the thin-film technique, instead of gluing on it a strain gauge laminated on polymeric foil, may provide a great improvement in sensor performance. A highly-sensitive and ultra-thin silicon stress sensor whose sensitivity is around 70 times that of metal strain gauge, are demonstrated flexible and sensitive enough for the measurement of mechanical strain on curved surfaces of human bodies (Zhao et al., 2014). Additionally, a novel flexible implantable device with higher sensitivities than those of commercial gauges has been applied for real-time mechanical strain measurement of chicken tibiae under three-point bending. Semiconductor strain sensors which are fabricated on flexible polyimide substrates, have significantly reduced sensor size and power consumption compared to metallic foil strain sensors and further decreased their effect on tissues’ deformation (Lisong et al., 2006).

To acquire immediate mechanical strain information of tissues *in vivo*, implantable and coated strain gauges with subminiature radio transmitter have been created. The telemetry measurement has been already verified available with the *in vivo* mechanical strain quantification of human cadaver spines during anteroposterior bending and torsion, though a small time shift occurred (Szivek et al., 2002). Whereas, all the elecro-based mechanical strain measurement techniques demand the insertion or attachment of sensors to target tissues and the soft tissues make it hard to manipulate. The application of wearable devices for the detection of real-time mechanical strain is becoming a potential method of health care.

The electro-based mechanical strain measurement, as an invasive method, determines the strain of tissues discretely and the strain it obtains is the average value of the measured regions. An investigation of the averaging effect showed limitations in adequate compensation and avoiding of the error magnitudes (Younis & Kang, 2011). Moreover, large deformation may produce damage of the circuit elements on rigid islands connected by stretchable wires, and the presence of rigid areas within the substrate limits its deformation. Though a wireless strain gauge was recently developed for remote mechanical strain measurement to eliminate the effect of wires, the range of measurable values decreased as the distance between the sensor and the reading unit increased (DiGiampaolo, DiCarlofelice & Gregori, 2017). Super thin substrate may provide a good solution (Sekitani et al., 2010). For curve mechanical strain measurement, fiber Bragg grating sensors were presented for the measurement of the bending curvatures of polyimide thin film skin with 48 sensors glued on the skin surface (Sun et al., 2018). Though mechanical strains at other regions can be calculated in conjunction with the cross-sectional area of the specimens, the computed values are probably different from those of actual strain distributions owing to the complex and heterogeneous structure in the samples. The accuracy of electro-based mechanical strain measurement techniques highly depends on the quality of strain sensors and their attachment or insertion to the tissues.

US-based methods are widely applied clinically as noninvasive techniques for mechanical strain measurement *in vivo*. Strong correlations were found between DIC and US radiofrequency elastographic estimates of mechanical strain (Chernak Slane & Thelen, 2014). Tissue Doppler imaging is highly angle-dependent and is for the mechanical strain testing of tissues in the direction parallel to US beam, while speckle-tracking echocardiography can track the tissue strain not restricted to the direction parallel to the US beam. In 2D speckle-tracking echocardiography, the tracked speckles may be missing in the tracking plane, which can be solved by 3D speckle-tracking echocardiography. The comparison of 2D speckle-tracking echocardiography and 3D speckle-tracking echocardiography has been reviewed already (Muraru et al., 2018), and the longitudinal and apical foetal speckle tracking echocardiography of the foetal heart in gestational woman was conducted for strain assessment with tissue motion annular displacement and segmental longitudinal strain (Derpa et al., 2018). Because US-based mechanical strain measurement techniques are built on US reflection, the mechanical strain of the tissues with complex and un-uniform structure can’t be evaluated precisely using the US-based mechanical strain measurement techniques.

MR-based methods can be utilized to test dynamic strain with a resolution of around 0.1 mm. For MRI, motion-induced phase shifts technique is with greater precision than the tag displacement measurement (Axel, 1997). No side effects have been found during the mechanical strain measurement with MRI, which indicates the latent ability for clinical use. However, due to the resolution of MRI, this technique has limitations in a small-scale local strain analysis. MR-based mechanical strain measurement is also expensive and time-consuming, which decreases its value as a clinical application.

The light-based mechanical strain measurement can only be used for superficial mechanical strain measurement or the mechanical strain measurement of transparent or translucent tissues due to the disability of optics to penetrate tissues. Their easy-operation, time-saving and low-cost prompt their applications in clinical practice and laboratories. A multi-camera speckle interferometer was lately developed and optimized for the full-field displacement measurement of human eye sclera during inflation testing (Bruno, Bianco & Fazio, 2018), suggesting the application of light interference.

In DIC, the imaging configurations play a vital role in the preciseness of mechanical strain measurement (Zhu et al., 2018), and the mechanical strain quantification is constricted to the superficial regions of the tissues. Tracking physical markers or speckles fixed on the sample surface is widely used to assess mechanical strain distribution on the tissue surface. Because of small size of the markers or speckles, less effect will be caused during the mechanical strain measurement. However, physical markers or speckles may move separately from the tissues and result in underestimated mechanical strain of the tissues (O’Connell et al., 2007). Nowadays, an increasing number of new techniques are arising. An intensity matching image registration method together with loaded and unloaded sequential micro-CT configurations have been developed and validated to measure mechanical strain fields in whole rat vertebrae (Hardisty & Whyne, 2009). With the technique development of mechanical strain measurement in biology, DVC has been extended to almost any imaging technology. DIC was applied to analyze the mechanical strain of rat Achilles tendons *ex vivo* using US images (Okotie et al., 2012). New methods with high resolution and speed should be developed with combined techniques including DVC and fast sequential micro-CT imaging for mechanical strain measurement in biological tissues. Color images are also proposed in DVC to improve the accuracy in the measurement of small mechanical strains, just as judging small strains in DIC (Hassan et al., 2016).

Recently, multi-scale mechanical strain measurement techniques have been utilized to provide a comprehensive overview of the mechanical strain evolution and distribution ranging from molecule-scale to tissue-scale mechanical strains. For example, synchrotron X-ray facilities have been used for tissue structure study in multiple length scales. When coupled with *in situ* loading, the techniques can be used to evaluate mechanical strains down to nanoscale. Imaging of these structures under tension (Bianchi et al., 2016; Gustafsson et al., 2018; Zimmermann et al., 2014), compression (Bergstrom et al., 2017; Dong, Almer & Wang, 2011) and bending (Karunaratne et al., 2012) can be determined for strain assessment. Gustafsson et al. combined global mechanical strain measurement with micro-scale tissue strain measurement and nano-scale collagen and mineral strain measurement in bovine cortical bone (Gustafsson et al., 2018). Similarly, an improved DIC was developed for the large strain measurement of cell substrate accurately (He et al., 2018), and the deformation of cells and their collagen constructs were assessed with scanning electron microscopy in a different study (Leung et al., 2018). In addition, the strains in deformed porcine meniscus from macroscale tissue to microscale cell were evaluated using DVC analysis of confocal microscopy images (Upton et al., 2008). Also, based on varying interactions between proteins in tissues and introduced peptides during the deformation of tissues, biochemical approaches with peptides were also conducted for the assessment of tissues’ mechanical strain (Arnoldini, 2017; Kubow et al., 2015).

Developing a real-time mechanical strain testing system that offers transient and high-quality images of mechanical strain fields is essential for clinical use. Combining different methods of mechanical strain measurement provides potential prospects. DVC, an image processing method, was applied to MR images for the quantification of intra-tissue strain fields of intra-tissue (Bey et al., 2002b). Confocal microscopy was used to track and capture images of fluorescently labeled cells in rat growth plates *in vitro* with applied mechanical strains and the local mechanical strain patterns were quantified using the DVC image processing approach (Villemure et al., 2007). In addition, a new system with the DVC image processing was developed and validated for direct internal mechanical strain measurement in connective tissues under controlled loads (Doehring, Kahelin & Vesely, 2009).

To reduce radiation dose and X-ray damage, extra effort should be given to design experiments for CT-based mechanical strain measurement both *in vivo* and *ex vivo*. Compared with MRI, X-ray CT mechanical strain examination is typically faster and is less prone to noise. With the evolution of testing techniques of mechanical strain, an algorithm, a mechanical strain estimation technique is presented to enhance the accuracy of mechanical strain evaluation, by directly estimating mechanical strain fields without previously first estimating displacements (Boyle et al., 2014). Another study showed that 2D image processing DIC offers the same mechanical strain accuracy as golden standard strain gauges under ideal conditions (Lee, Take & Hoult, 2012), and pointwise correlation algorithm DIC showed better mechanical strain preciseness than the traditional subset-based correlation algorithm DIC (Jin, 2005). First-order algorithm could significantly reduce strain measurement error, with MR images of canine knee menisci and porcine intervertebral discs (Gilchrist et al., 2004). Moreover, an iterative non-linear curve-fitting algorithm was introduced to test rat myocardium mechanical strain with 3D high-frequency US speckle tracking (Yap et al., 2015).

In conclusion, besides electro-based mechanical strain measurement techniques, almost all the other techniques for the evaluation of mechanical strains in tissues are based on the imaging of the tissues and analysis algorithms for the mechanical strain calculation from the images. Thus, combining and developing DVC with imaging techniques including high-speed and universal X-ray micro-CT modalities is a promising way for 3D mechanical strain evaluation of tissues.

## Additional Information and Declarations

### Data Availability

The following information was supplied regarding data availability: This article is a literature review and therefore did not generate, collect or analyze any raw data or code.
